# Communication medium in theileriosis control: the factors that determine disease knowledge among smallholder farmers in Zimbabwe

**DOI:** 10.1007/s11250-023-03466-x

**Published:** 2023-02-16

**Authors:** Shelton M. Nhokwara, Hiroichi Kono, Satoko Kubota, Mark Jubenkanda

**Affiliations:** 1 Doctor Program of Animal Science and Agriculture - Obihiro University of Agriculture and Veterinary Medicine, Obihiro, Hokkaido Japan; 2 Department of Agro-environmental Science, Obihiro University of Agriculture and Veterinary Medicine, Hokkaido Obihiro, Japan; 3 Department of Veterinary Services, Ministry of Lands, Agriculture, Fisheries, Water, Climate and Rural Development, Harare, Zimbabwe

**Keywords:** Communication, Theileriosis, Knowledge, Zimbabwe

## Abstract

Theileriosis is one of the most important tick-borne diseases that has been affecting farmers and thousands of livestock in Zimbabwe. The main government strategy to combat theileriosis is use of plunge dips with anti-tick chemicals at specified times; however, an increase in number of farmers caused a strain on government services resulting in disease outbreak. One of the key issues that have been highlighted by department of veterinary is the strain in communication and knowledge of the disease with the farmers. Hence, it is important to evaluate the communication between farmers and veterinary services and identify possible areas of strain. A field survey was conducted with 320 farmers in Mhondoro Ngezi, a district badly affected by theileriosis. Face-to-face interviews with smallholders and communal farmers were conducted between September and October 2021, and the data were analyzed using Stata 17. Communal farmers relied mainly on oral communication and had limited knowledge of theileriosis; therefore, dead cattle % was high among them. Though veterinary extension officers were the prime source of information, oral communication medium affected knowledge transferred. The results of this study recommend adoption of communication mediums that encourage retention, such as brochures and posters by veterinary extension services. The government may also partner with private players to ease pressure of increased farming population due to land reform.

## Introduction

### Farmer knowledge

Farmer knowledge is essential for developing agriculture and increasing productivity (Janc et al. 2012). In veterinary extension, farmer knowledge includes understanding animal health practices and methods to prevent and contain diseases. In animal health, knowledge is determined by the ability to know the cause of the disease, tell the symptoms, and know the remedies (Ducrot et al. 2011a, b). Building farmer knowledge can be possible when there is effective communication (Adamsone-Fiskovica and Grivins 2021). Investing in communication within agriculture increases farmer empowerment (Faqih and Aisyah 2016). Agricultural communication prioritized is only a stimulus for the adoption of technologies, and not a complete means of change (Balamatti and Biradar 2016; Moyo and Salawu 2018); thus, communication is essential in building farmer knowledge. In Zimbabwe, the Agricultural, Technical, and Extension Services (AGRITEX) is responsible for government to farmer communication, while the Department of Veterinary and Extension Services (DVS) is responsible for communication on animal production (Sungirai et al. 2015). Two key communication pillars are source of information and the medium used, and they can influence public perception and behavior (Wilkins et al. 2018). Increasing farmer knowledge depends on efficient communication and enough technical support.

In Zimbabwe livestock diseases are prioritized because of the importance of livestock in production, transport, meat and milk supplies, storage of wealth, and generation of household income (Tavirimirwa et al. 2013), with overall contribution of 35–38% of the Gross Domestic Product (GDP) of the Agricultural sector which contributes 17% to overall GDP (FAO 2020). The contribution to the economy and to individual farmers is threatened diseases like foot and mouth disease and secondly by tick-borne diseases, chief among them bovine theileriosis (Manyenyeka et al. 2021; The Patriot 2022).

Theileriosis is an animal disease caused by members of the protozoal genus *Theileria* (Lawrence and Waniwa 2020) mainly affecting cattle in eastern, central, and southern Africa. Its mitigation has relied on effective control of the tick vector *Rhipicephalus appendiculatus*. In Zimbabwe, it is known as “January disease” and specifically a disease of cattle caused by *Theileria parva* (Lawrence and Waniwa 2020). *January disease* is the name used because the disease is highly transmitted at the peak of rainy season in January. Signs of theileriosis include breathlessness, poor appetite, swollen lymph nodes, low milk production, and high temperature, among others (Lawrence and Waniwa 2020). In East Africa, the Muguga vaccine is used (Allan and Peters 2021), while in Zimbabwe, currently cattle dipping is main control method (Muvhuringi et al. 2022).

Cattle dipping, weekly and fortnightly in summers and winter, respectively, is the main control strategy in Zimbabwe (Muvhuringi et al. 2022; Sungirai et al. 2018). Cattle are dipped in a plunge dip filled with water containing chemical to break the life cycle of ticks, and during outbreaks, the 5–5-4 dipping strategy is recommended by DVS (Muvhuringi et al. 2022). The 5–5-4 dipping method means cattle dipping twice for 5 days, followed by a 4-day interval to break the life cycle of the tick, because of the short tick engorgement period in wet environments (Muvhuringi et al. 2022; Sekkin 2017; Walker 2011). However, there is no recorded data showing with statistics of the effectiveness of the 5–5-4 dipping method, while some attribute its effectiveness to low cases of the disease in other parts of the country. Disease control initiatives by the government have mainly been technical; consequently, tick-borne disease outbreaks have always continued. The 2018–2021 period recorded a year on year increase in both cases of theileriosis and deaths of cattle claiming at least 50,000 cattle (Muvhuringi et al. 2022; New Zimbabwe 2022; Lawrence and Waniwa 2020); this was not the trend in previous years as Zimbabwe was able to export meat because of disease control.

The main affected groups are smallholder and communal farmers who occupy 96% of the agriculture land and account for 90% of the cattle (Zimfact 2018) and are the main beneficiaries of the fast track land redistribution which tilted land ownership in favor of rural population (Sungirai et al. 2015). This led to a shift in cattle ownership and ultimately resulting in constrain to the veterinary services affecting communication and service delivery (DVS 2022). The communal farmers group is reported to be not fully participating in tick control activities, yet owning more cattle (DVS 2022), indicating that there exist gaps in communication and knowledge of theileriosis among farmers which may have resulted in increase in cattle deaths.

In Africa farmer knowledge is mainly affected and/or determined by socio-economic factors, and this varies by country (Mutimura et al. 2018). In rural Kenya, farmer knowledge was largely affected by household head age and education among other factors (Mogaka et al. 2021); communication was considered part of the key solutions to closing the knowledge gap. While studies conducted in Zimbabwe have focused on accounting the tick species, epidemiology of tick diseases, and case locations (Manyenyeka et al. 2021; Sungirai et al. 2015; Latif et al. 2001), little attention has been paid on socio-economic factors that determine farmer knowledge of theileriosis in Zimbabwe and how it impact cattle death. Furthermore, research conducted in Zimbabwe showed that farmers lack knowledge and suggests communication would be necessary to increase knowledge, reduce disease prevalence, and increase productivity (Thomas and Babu 2020; Musungwini 2018).

### Objective of the research

In recent years, much research has focused on impact of theileriosis in Zimbabwe (Moyo et al 2017; Manyenyeka et al. 2021), but with the increase in knowledge of the disease and ticks, there is little focus on farmer characteristics as determinants of knowledge among the different farmer groups in Zimbabwe and how communication is used. Purpose of this research was to also establish socio-economic determinants of knowledge of theileriosis in smallholder and communal farmers in Mhondoro Ngezi. This study aimed to fill the gap in the impact of communication on theileriosis knowledge and the effect on reducing dead cattle among communal and smallholder farmers.

## Methodology

This study was conducted between September 2021 and October 2021 in Mhondoro Ngezi District, Mashonaland West Province, Zimbabwe. The area spanning 9.327 ${\mathrm{km}}^{2}$ is in farming region 3, recording 650–800 mm rainfall annually. It contains 16 wards and a farmer population of 6077. Mashonaland West Province was selected for this study because it recorded the highest number of cattle deaths due to theileriosis between 2019 and 2021. Multistage sampling technique was adopted to select respondents; the first stage was selection of Mhondoro Ngezi district, wherein stratified sampling was performed among smallholders and communal landholders. Systematic random sampling techniques were used to select farmers per dip tank listed from six selected dip tanks in the district. Three hundred twenty farmers were selected using the formula *n*=${z}^{2}pq/{e}^{2}$ (Kothari 2004). Here, *n* = sample size, *z* = standard normal deviate set at 1.96, corresponding to a 95% confidence interval, *p* = proportion of livestock farmers in Mhondoro Ngezi, *q* = 1-p, and *e* = maximum allowable error 0.05.

Data were collected using a structured questionnaire for individual farmers through face-to-face interviews, and open-ended questionnaire was used for focus groups comprising five to seven farmers. The questionnaire was pre-tested on five farmers drawn from different dip tanks. Pre-test was limited to a few farmers in the sample size because of the COVID-19 protocols and restrictions on movement. Questionnaires were administered by veterinary extension officers from DVS in Mhondoro Ngezi, who were tasked with collecting data in an area they did not supervise, to remove bias. The data were analyzed in Stata 17 using a Tobit model with endogenous regressor.

### Conceptual model

An empirical challenge exists in establishing a causal relationship (Akter et al. 2020) between theileriosis knowledge and dead cattle % which means endogeneity exists. Farmers may not have the liberty of selecting the communication medium used as there is a strain in the veterinary extension services. This means that the dead cattle % can be affected by other unobserved factors; thus, the endogeneity of dead cattle % was associated with not only changes in variable like veterinary access, tick control methods, grazing system, and frequency of access but also changes in the error term. To correct this, the instrumental variable estimation was selected for establishing the causal effect of communication on dead cattle %. As there are other variables that do not directly affect dead cattle %, an analysis of their relationship with theileriosis knowledge was assessed using instrumental variable method. Instrumental variable method, a two-stage least square technique used to estimate relationships of interest (Angrist and Krueger 2001), was used to estimate relationship between increasing theileriosis knowledge and dead cattle %. Dead cattle % was the dependent variable. The independent variables were socio-economic in nature; these are landholding, age, ${age}^{2}$, sex, income source, cattle herding experience, education, veterinary access, and cattle feeding system. The socio-economic variables were used basing on previous research suggesting that rural farmer knowledge in developing countries is mainly affected by issues like age, household income, and education, among others (Mogaka et al. 2021; Erminio 2019; Mafimisebi et al. 2012). The endogenous variable was *theileriosis knowledge*, while instruments were *information source* and *communication medium*.

The purpose of using instrument variables was to measure how knowledge of theileriosis is influenced by source of information and communication medium and how it impacts dead cattle %. The instruments used meet the condition of the instrumental variable because the instruments are exogenous (Angrist et al. 1996), as both information source and communication medium cannot be determined by the farmers but mainly by veterinary.

### Econometric model

The first stage regression equation can be presented as:

$${Theileriosis\;knowledge}_{{\varvec{i}}}={{\varvec{\delta}}}_{0}+{{\varvec{\delta}}}_{1}\cdot Information\;{Source }_{{\varvec{i}}}+{{\varvec{\delta}}}_{2}\cdot\;{Communication\;medium}_{{\varvec{i}}}+{{\varvec{\delta}}}_{3}\cdot {Land\;holding}_{{\varvec{i}}}+{{\varvec{\delta}}}_{4}\cdot\;Farmer\boldsymbol{\;}{Age}_{{\varvec{i}}}+{{\varvec{\delta}}}_{5}\cdot\;Farmer\boldsymbol{\;}{{Age}^{2}}_{{\varvec{i}}}+{{\varvec{\delta}}}_{6}\cdot Farmer\boldsymbol{\;}{Sex}_{{\varvec{i}}}+{{\varvec{\delta}}}_{7}\cdot\;Income\;{Source}_{{\varvec{i}}}+{{\varvec{\delta}}}_{8}\cdot\;Cattle\;herding\boldsymbol{\;}{experience}_{{\varvec{i}}}+{{\varvec{\delta}}}_{9}\cdot {Education}_{{\varvec{i}}}+{{\varvec{\delta}}}_{10}\cdot\;Veterinary\boldsymbol{\;}{Access\;Time}_{{\varvec{i}}}+{{\varvec{\delta}}}_{11}\cdot\;Feeding\boldsymbol{\;}{System}_{{\varvec{i}}}+{{\varvec{u}}}_{{\varvec{i}}}$$

The second stage regression equation can be presented as:

$$Dead\;cattle \% = {{\varvec{\gamma}}}_{0}+{{\varvec{\gamma}}}_{1}\boldsymbol{\;}Theileriosis\;knowledge+{{\varvec{\gamma}}}_{2}\cdot {Land\;holding}_{{\varvec{i}}}+{{\varvec{\gamma}}}_{3}\cdot\;Farmer\boldsymbol{\;}{Age}_{{\varvec{i}}}+{{\varvec{\gamma}}}_{4}\cdot\;Farmer\boldsymbol{\;}{{Age}^{2}}_{{\varvec{i}}}+{{\varvec{\gamma}}}_{5}\cdot\;Farmer\boldsymbol{\;}{Sex}_{{\varvec{i}}}+{{\varvec{\gamma}}}_{6}\cdot\;Income\;{Source}_{{\varvec{i}}}+{{\varvec{\gamma}}}_{7}\cdot\;Cattle\;herding\boldsymbol{\;}{experience}_{{\varvec{i}}}+{{\varvec{\gamma}}}_{8}\cdot {Education}_{{\varvec{i}}}+{{\varvec{\gamma}}}_{9}\cdot\;Veterinary\boldsymbol{\;}{Access\;Time}_{{\varvec{i}}}+{{\varvec{\gamma}}}_{10}\cdot Feeding\boldsymbol{\;}{System}_{{\varvec{i}}}+{\mathcal{E}}_{{\varvec{i}}}$$

Table 1 summarizes the description of the variables used in this study and mean findings of the data.

**Table 1:** Description of variables

Variable	Definition	Mean	Standard deviation
Dead cattle %	Percentage of dead cattle in farmers’ present herd	0.44	0.31
Knowledge of theileriosis	1 = Have knowledge about theileriosis symptoms, cause, and prevention 0 = No knowledge about theileriosis	0.76	0.42
Landholding	1 = Smallholder farmers 0 = Communal farmers	0.32	0.46
Age	Farmer age in years	56	14.80
Sex	1 = Male 0 = Female	0.88	0.31
Income source	1 = Income from livestock sales and milk 0 = No income from livestock sales and milk	0.43	0.49
Cattle herder experience	Years working in livestock farming	25	17.71
Education	1 = Basic education 0 = No basic education	0.06	0.24
Vet access time	1 = Monthly access to veterinary services 0 = No monthly access to veterinary services	0.42	0.02
Feeding system	1 = Community land 0 = No community land	0.98	0.11
Information source	1 = Information from veterinary office 0 = Information from social network	0.65	0.47
Communication medium	1 = Oral communication 0 = Written communication	0.91	0.28

## Study results

### Theileriosis knowledge

This research showed that 76% of the farmers had knowledge of the disease and 94% of the farmers could identify at least one major symptom especially swollen lymph nodes. The other symptoms are acceptably known, and some, such as low milk production and yellow gums, are barely known, as depicted in Table 2. Differences in statistics show that there is a gap in the levels of knowledge between the smallholder farmers and communal farmers, with the communal farmers being less-knowledgeable about theileriosis symptoms.

**Table 2:** Knowledge of the symptoms of theileriosis

Symptoms	% in smallholder farmers (*n* = 107)	% in communal farmers (*n* = 213)
Cloudiness of eyes	39	74***
Swollen lymph nodes	33	44
Low milk production	1	6
Shortness of breath	59	56
Loss of appetite	95	94
Staggering	18	35***
Yellow gums	14	4
Death	15	33
Moving behind	53***	32

# ***, significance 1% (*n* = 320)

We established that there is difference in knowledge levels if the farmer knows the disease, but unable to tell symptoms, preventative techniques, and control methods. To distinguish this clearly, the researchers also focused on differences in knowledge between smallholder and communal farmers.

**Fig. 1:**
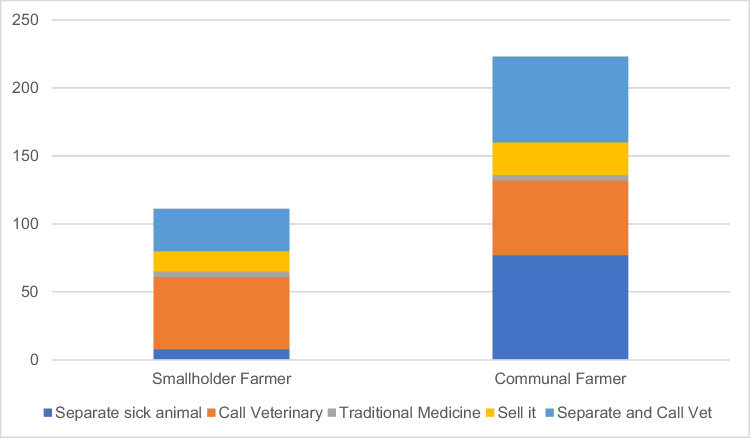
Preventative techniques (*n* = 320)

Preventative techniques are aimed at reducing spread within and between cattle herds. Smallholder farmers relied more on calling veterinary officers and separating sick animals. However, communal farmers relied not only on separating the sick cattle and calling the vet, but also selling the cow to recover the value which can cause health disasters. Figure 1 presents the statistics from the research, showing both the category and techniques used by each farmer type.

**Fig. 2:**
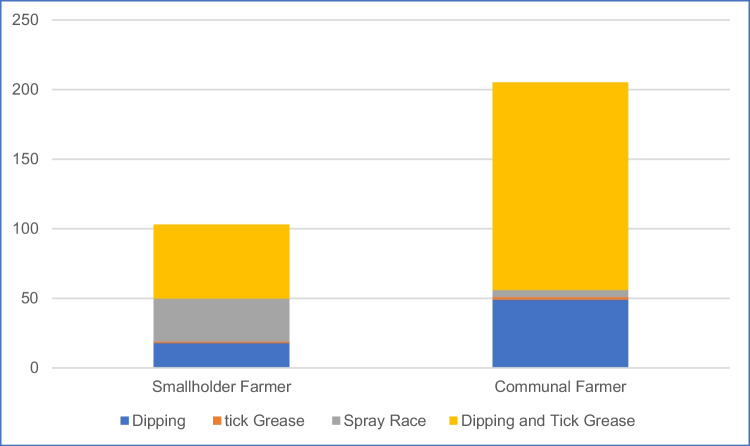
Tick control method (*n* = 320)

The tick control methods used in this study included dipping cattle, applying tick grease, using a spray race, and a combination of dipping and tick grease. Communal farmers used the dipping strategy and the combined dipping and tick grease technique, which are services provided mainly by the government. However, with DVS limited resources, the communal farmers suffer. In contrast, smallholder farmers used government services as well as the spray can, a self-made method, to eliminate ticks which seemed effective to reduce cattle deaths. Figure 2 illustrates these results.

**Fig. 3:**
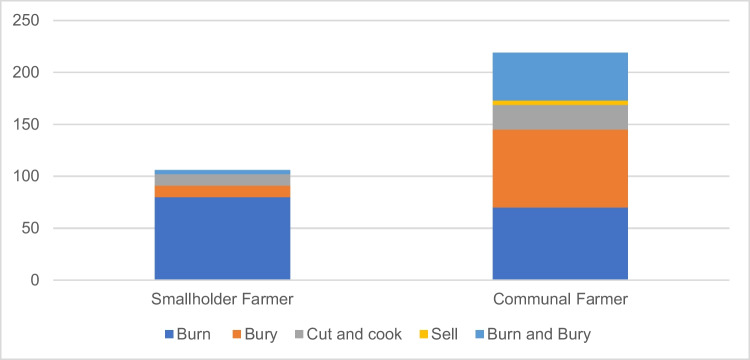
Disposal of dead cattle (*n* = 320)

*Theileriosis knowledge* cannot be assessed on its own; additional questions were asked to the farmers regarding disposal of dead cattle, as shown in Fig. 3. The recommended disposal method is to burn the cattle. Smallholder farmers followed the disposal recommendations, while some communal farmers buried the dead cattle, sold the dead cow, or prepared the meat for cooking.

### Factors affecting theileriosis knowledge

*Landholding*, *farmer age*, and *education* had significant positive relationships with knowledge of theileriosis at 1% significance level, as shown in Table 3. As age increases, chances to gain more theileriosis knowledge increase until a certain age, and the ${Farmer Age}^{2}$ shows that as age increases, knowledge decreases. *Cattle herder experience* and *veterinary access time* had negative relationships with knowledge of theileriosis at 1% significance level.

**Table 3:** Instrumental variable regression on dead cattle % and theileriosis knowledge

Variables	(A) Knowledge of theileriosis		(B) Dead cattle %	
	Coefficient	*P* >|z|	Coefficient	*P* >|z|
*Knowledge of theileriosis*	-	-	− 1296.846	0.005***
*Landholding*	0.1811343	0.000***	14.98209	0.893
*Farmer age*	0.0260521	0.004***	− 2.911411	0.897
${Farmer Age}^{2}$	− 0.0002429	0.005***	0.0479316	0.830
*Farmer sex*	− 0.0544862	0.362	20.06153	0.835
*Income source*	− 0.2022601	0.000***	− 270.3367	0.073*
*Cattle herder experience*	− 0.1839945	0.000***	0.693127	0.994
*Education*	0.2138603	0.003***	195.8873	0.235
*Veterinary access time*	− 0.2481961	0.000***	− 357.2864	0.015**
*Feeding system*	− 0.2003448	0.010**	− 433.8759	0.034**
*Information source*	0.1063687	0.041**	-	-
*Communication medium*	− 0.1927319	0.000***	-	-
_cons	0.8430071	0.000	1965.422	0.006
Observation WaldChi2 (13)	318 37.27***		318 37.27***	

# ***, **, and * indicate significance at the 1%, 5%, and 10% levels, respectively

### Factors affecting dead cattle %

The second-stage regression aimed to understand the variables that can affect the number of dead cattle. The first instrument is the extraction from the first-stage regression, which shows that there is a negative relationship between the *knowledge of theileriosis* and the percentage of dead cattle at 1% significance level. Farmers with agro-based *income sources* relying on animal sales and milk had low dead cattle %. Significant relationship at 10% level indicates that the more farmers engaged in livestock-related income, the more they protected the cattle herd from diseases, the less cattle died. Increased *veterinary access time* showed a significant relationship with dead cattle %.

The *information source* is critical as it denotes how a farmer obtains information about animal health. The variable *information source* had a 5% significance level and was positively related to theileriosis knowledge. This indicates that farmers who had access to veterinary extension officer as source of information were more knowledgeable than those who did not have access. There is a possibility of increasing the level of knowledge of theileriosis if the DVS as the custodian of animal health and surveillance is more active in disseminating information regarding animal health. The use of official sources of information is key to managing the knowledge transmitted to the farmers; however, in the context of Zimbabwe, the strain on veterinary officers, due to an increase in the farmer population, calls for new ways of communicating with farmers, which may include using technology and mass media.

*Communication medium* had a negative relationship with the knowledge of the disease at 1% significance. This is because the use of oral communication to pass information had no lasting impact on the farmers’ theileriosis knowledge. Absence of written communication can be due to a number of reasons, spanning from illiteracy of farmers to financial constraints of the government in producing written communication even in local language. The use of oral communication could have been necessitated by the fact that farmers have limited exposure to writing as a form of communication, given that their education is mainly between the primary and secondary levels, especially in communal areas, while also the government may have limited resources to provide written information. This claim is supported by the UNICEF’s 2021 report, which highlighted that secondary school education rate in Zimbabwe is very low reporting high attendance of secondary school and low completion. This will, in turn, impact the level of knowledge these farmers accumulate, as well as the communication medium they use to acquire knowledge.

## Discussion

We study the socio-economic determinants of theileriosis knowledge among smallholder and communal farmers as influenced by communication used by the veterinarian. The first characteristic was landholding. More farmers were in the communal area, while the percentage of dead cattle was higher for the farmers in the communal area. This means that farmers in smallholder category have higher chance of gaining knowledge about theileriosis than the farmers in communal areas; thus, they had low dead cattle percentage. These findings are supported by Obidike (2011) who highlights that rural farmers have less access to knowledge and at times rely on traditional knowledge.

Farmers who have a basic level of education tend to gain more knowledge about theileriosis than farmers who have no educational background. These results agree with the statistical information that despite the high literacy rate in Zimbabwe, the level of education attendance is low in rural areas (Shoko 2014). A UNICEF (2021) report shows that primary and high-school dropouts are common in rural areas of Zimbabwe and that the completion rate for ordinary level education is very low. Higher education can influence the adoption of new farming methods and improve farm productivity (Warmbrod 2019; Paltasingh and Goyari 2018). These research findings agree with those of studies on agricultural productivity and education conducted in India and parts of Africa. An emphasis on increasing agricultural education can help disseminate more knowledge about theileriosis in Zimbabwe. There is a need to focus on community training programs for disease awareness and to increase livestock education among those in the school-going age group by engaging veterinary officers in school education as some cattle herders are kids of school going age.

This indicates that the increase in experience affects the accumulation of theileriosis knowledge, considering the average years a farmer has dealt with cattle is 20 years. Farmers who have limited access to veterinary services over the course of a month have limited theileriosis knowledge. Presumably, farmers who have more contact with veterinary services monthly have more knowledge of animal health. Additionally, farmers with fewer years of cattle experience tend to know more about theileriosis because young farmers are receptive to new knowledge and information. Akter et al. (2020) highlights that smallholder farmers who adopt to technology improve agriculture productivity; thus, if the famer population be more old aged adoption of technologies is low. This is also supported by Musungwini (2018), on a study in Zimbabwe that information asymmetry in agriculture can be solved by introducing technology, but the older the farmer, the less the technology uptake which means younger farmers are to be supported to be active in agriculture.

Increase in knowledge of theileriosis will result in significant decrease in dead cows. Thomas and Babu (2020) highlighted that increasing farmer knowledge will improve agricultural productivity, thus supporting our finding. In the current study, the smallholders were more knowledgeable and had a lower percentage of dead cattle compared with communal farmers. This means that the group with the largest cattle ownership has limited knowledge which will result in the total cattle population in Zimbabwe being affected. Therefore, it is necessary to manage the knowledge transfer among communal farmers so as to preserve the national herd.

Higher frequency of access to veterinary services resulted in reduction in dead cattle. Farmers with low access to vet services reported more dead cattle. Farmers in smallholder were more likely to call a veterinarian than communal farmers who relied more on separating the sick cows from the unaffected cows. Pahwa and Swain (2020) showed that there is need for frequent communication, and educating rural farmers helps reduce the effect of zoonotic diseases. The findings of our study agree with those of the above-mentioned study in showing that there is a need to frequently educate farmers to reduce the spread of theileriosis.

## Conclusion

According to this study, increase in incidence of dead cattle can be attributed to the limited knowledge of theileriosis among farmers, mainly in communal farmer category due to communication issues in veterinary extension. Communal farmers have more years of cattle herding experience and an aging farmer population, both of which have negative effects on gaining knowledge. Relying on an aged population has negative effects to the advancement of agriculture in Zimbabwe; thus, the government must put in place policies that empower the younger generation to be active in agriculture and support their activities. Educational curriculum can be inclusive of livestock farming to groom upcoming generations of farmers.

Farmers in the smallholder category could identify more signs of theileriosis and used more tick control methods that relied less on government services and had more income related to agriculture. This enabled smallholders to manage the disease quickly and effectively, resulting in fewer theileriosis-related deaths among the cattle. Communal farmers need more access both markets and information, and this will mean more participation in the agriculture value chain thereby reducing the probability of poverty. Policies that promote the participation of communal farmers in livestock value chain to encourage them to be economically active must be considered despite of the landholding. The government should increase land access to young farmers who are keen to learn new things in animal husbandry and other agriculture technologies.

The farmers obtained most of their information from correct source, the veterinary office, but the access times and communication media used have been important factors. Communication medium that promotes record-keeping and veterinary access times with short intervals between visits are important to promote knowledge of theileriosis. The farmers had limited knowledge because of the infrequent access to veterinary services, and oral communication affected information retention for reference purposes when the veterinary services were inaccessible due to issues like communication network or even road access. The government must promote frequent communication by veterinary extension officers and help improve the communication by enabling accessibility to the farmers and availability of information in written form, which could include simple posters in the local language or the distribution of dipping calendars with relevant dip-cycle information.

Furthermore, the government must encourage private players such as veterinary distributors, who frequently communicate with the farmers on other issues to be conduits of government campaign on disease awareness. Collaboration with private players can help improve communication channel and mediums used, thus improving farmer knowledge levels and help in reducing disease prevalence.

This research serves to fulfill part of the strategy by the DVS Zimbabwe on the Integrated Tick and Tick-Borne Disease Control Strategy 2022–2030, aimed at eradicating the disease and improving the cattle herd in Zimbabwe.

## Data Availability

All data used in this research is accessible through request to the corresponding author.

## Code availability

The analysis of data was done in Stata 17, Licensed to: Shelton, under the Obihiro University of Agriculture and Veterinary Medicine, and the code is available upon request to the corresponding author.

## References

[CR1] Anda Adamsone-Fiskovica & Mikelis Grivins, 2021, Knowledge production and communication in on-farm demonstrations: putting farmer participatory research and extension into practice, The Journal of Agricultural Education and Extension Competence for Rural Innovation and Transformation, Volume 28, 2022 - Issue 4

[CR2] Akter Sonia, Chindarkar Namrata, Erskine William, Spyckerelle Luc, Imron Julie (2021). Lucia Viana Branco, 2020, Increasing smallholder farmers’ market participation through technology adoption in rural Timor-Leste, Asia & the Pacific. Policy Studies.

[CR3] Allan FK, Peters AR (2021). Safety and Efficacy of the East Coast Fever Muguga Cocktail Vaccine: A Systematic Review. Vaccines (basel)..

[CR4] Angrist J D, Imbens G W, Rubin D B 1996 Identification of causal effects using instrumental variables. Journal of the American Statistical Association

[CR5] Angrist JD, Krueger AB (2001). Instrumental Variables and the Search for Identification: From Supply and Demand to Natural Experiments. Journal of Economic Perspectives.

[CR6] Arun Balamatti and Nagaratna Biradar, 2016 Modern Media in Agricultural Communication, Conference Paper Lead paper submitted to the 8th GCRA International Conference on “Innovative Digital Applications for Sustainable Development”, 5 – 7, January 2016, UAS, Bengaluru.

[CR7] Horace G. Campbell, 2007, The Zimbabwean Working Peoples and the Land Question, pp. 23–31 (9 pages) Published By: Taylor & Francis, Ltd.

[CR8] Deloitte & Touche, 2015, Reducing Food Loss Along African Agricultural Value Chains

[CR9] Department of Veterinary Services Zimbabwe, 2022, Zimbabwe Integrated Tick and Tick-Borne Disease Control Strategy 2022-2030

[CR10] Ducrot C, Bed'Hom B, Béringue V (2011). Issues and special features of animal health research. Vet Res.

[CR11] Christian Ducrot, Bertrand Bed'Hom, Vincent Béringue, Jean-Baptiste Coulon, Christine Fourichon, Jean-Luc Guérin, Stéphane Krebs, Pascal Rainard, Isabelle Schwartz-Cornil, Didier Torny, Muriel Vayssier-Taussat, Stephan Zientara, Etienne Zundel & Thierry Pineau , 2011a, Issues and special features of animal health research, *Veterinary Research* 42, Article number: 96 (2011a) 10.1186/1297-9716-42-96 PMC3170600 21864344

[CR12] Longhini Francesco Erminio, 2019, Communicating agricultural model concepts and results to smallholder farmers in rural areas of southern Zimbabwe: sharing knowledge for a mutual benefit, MSc Thesis Wageningen University and Research, 71 p.

[CR13] FAO (Food and Agriculture Organization). 2020. Zimbabwe at glance. Online. Available at http://www.fao.org/zimbabwe/fao-in-zimbabwe/zimbabwe-at-a-glance/en/

[CR14] A Faqih and S Aisyah, 2016 Communication in agricultural extension services toward farmer empowerment Journal of Physics: Conference Series, Volume 1360, International Symposium on Sciences, Engineering, and Technology 19–20 November 2018, Cirebon, Indonesia

[CR15] Janc Krzysztof, Czapiewski Konrad, Floriańczyk Zbigniew (2012). The importance and diffusion of knowledge in the agricultural sector: The Polish experiences. Geographia Polonica.

[CR16] Kothari, C. R., 2004 Research methodology: Methods and techniques (2nd ed.). New Age International Pvt Limited.

[CR17] Latif AA, Hove T, Kanhai GK, Masaka S, Pegram RG (2001). Epidemiological observations of Zimbabwean theileriosis: Disease incidence and pathogenicity in susceptible cattle during Rhipicephalus appendiculatus nymphal and adult seasonal activity. Onderstepoort Journal of Veterinary Research.

[CR18] Lawrence, J. A., & Waniwa, E., 2020. Theileriosis Today: A National Crisis. http://www.cfuzim.com/wp-content/uploads/2019/11/theileriosis.pdf

[CR19] Mabaye Tapiwa M., 2005 An Examination of Past & Present Policy, Shortcomings & Successes and Recommendations for Improvement, Paper for Conference

[CR20] Wijerathna Madhavi and Wanigasundera A D P, 2020b, “Communication” in the Context of Agricultural Extension: Past, Present and Way Forward in Achieving Sustainable Food Systems in Sri Lanka, In book: Agricultural Research for Sustainable Food Systems in Sri Lanka Publisher: Springer, Singapore

[CR21] Mafimisebi TE, Oguntade AE, Fajemisin AN (2012). Local knowledge and socio-economic determinants of traditional medicines' utilization in livestock health management in Southwest Nigeria. J Ethnobiology Ethnomedicine.

[CR22] Musaemura Manyenyeka, Whatmore Munetsi Tagwireyi, Munyaradzi Christopher Marufu, Reverend Moregood Spargo, Eric Etter; 2021; Spatio-temporal clustering and risk factor analysis of bovine theileriosis (Theileria parva) in Zimbabwe from 1995 to 2018, Wiley online Library; 10.1111/tbed.14081 10.1111/tbed.14081 33750039

[CR23] Masendeke, A., Kamuzhanje, J., Sithole, R., 2010. Participatory Extension Approaches in Zimbabwe. Ministry of Agriculture. Mechanisation and Irrigation Development Practical Action Southern Africa, Harare

[CR24] Warmbrod Matt, 2019 Discussing the problems with poor infrastructure and education, along with some proven solutions to increase productivity, www. arcgis.com

[CR25] Ministry of Lands, Agriculture, Water, Fisheries & Rural Resettlement. (2020). Second round crop and live-stock assessment report 2019/2020 season.

[CR26] Minjauw B, McLeod A., 2003 Tick-borne diseases and poverty. The impact of ticks and tick-borne diseases on the livelihood and marginal livestock owners in India and Eastern and Southern Africa Research report, DFID Animal Health Programme, Centre Of Tropical Veterinary Medicine, University of Edinburgh

[CR27] Mogaka BO, Bett HK, Ng’ang’ SK (2021). Socio-economic factors influencing the choice of climate-smart soil practices among farmers in western Kenya. Journal of Agriculture and Food Research.

[CR28] Moyo IA, Mudimba TN, Ndhlovu DN, Dhliwayo S, Chikerema SM, Matope G (2017). Temporal and spatial patterns of theileriosis in Zimbabwe: 2000–2014. Bulletin of Animal Health and Production in Africa.

[CR29] Moyo, Rachel and Salawu, Abiodun, 2018, A survey of communication media preferred by smallholder farmers in the Gweru District of Zimbabwe, Journal of Rural Studies

[CR30] Musungwini, Samuel, 2018 Mobile Phone Use by Zimbabwean Smallholder Farmers: A Baseline Study. *AJIC* vol.22, pp.29–52. ISSN 2077–7213. 10.23962/10539/26171.

[CR31] Mupenzi Mutimura, Paul Guthiga, Ruth Haug, Nigussie Dechassa, Mengistu Ketema, Feyisa Hundessa, Bosena Tegegne, Kibebew Kibret, Tamado Tana, Alice Murage, George Nyamu, Mercy Mburu, Mangani Katundu, Victoria Ndolo, Jacqueline Tuyisenge, Leonidas Dusengemungu, Eugenie Nyiransengimana, Myeni, L., Moeletsi M.E., Mphephu, M., Modiselle S, Kenneth Mapunda, Ahamad K. Athman, Ismail Selemani, Dismas Mwaseba, 2018, Socio-economic status affecting smallholder farming and food security with a focus on rural youth in Africa,

[CR32] Obidike, N.A. (2011) Rural Farmers’ Problems Accessing Agricultural Information: A Case Study of Nsukka Local Government Area of Enugu State, Nigeria. Library Philosophy and Practice

[CR33] Muvhuringi Prosper Bright, Murisa Rutendo, Sylvester Deliwe, Chigede Ngavaite, Mafunga Kudakwashe (2022). Factors worsening tick borne diseases occurrence in rural communities. A Case of Bindura District, Zimbabwe, Cogent Food & Agriculture.

[CR34] Pahwa S, Swain S (2020). The fate and management of sick and dying cattle – Consequences on small-scale dairy farmers of peri- urban areas in India. Indian J Community Med.

[CR35] Paltasingh KR, Goyari P (2018). Impact of farmer education on farm productivity under varying technologies: case of paddy growers in India. Agric Econ.

[CR36] Sekkin, S, 2017. Livestock Science. Intech Open. 9789535128649. 9789535128649 https://www.intechopen.com/books/5345, Chapter 7, Bacterial Tick-Borne Diseases of Livestock Animals

[CR37] Janet Shoko, 2014, Latest Zimbabwe education statistics embarrass ruling party www.theafricareport.com 19 May, 2014

[CR38] Sungirai, M., Moyo, D. Z., De Clercq, P., & Madder, M., 2016. Communal farmers’ perceptions of tick-borne diseases affecting cattle and investigation of tick control methods practiced in Zimbabwe. Ticks and Tick-Borne Diseases, 7, 1–9. https://www-sciencedirect-com.uplib.idm.oclc.org/science/article/pii/S1877959X15001454 10.1016/j.ttbdis.2015.07.015 26234572

[CR39] Sungirai, M., Moyo, D. Z., De Clercq, P., Madder, M., Vanwambeke, S. O., and De Clercq, E. M. (2018). Modelling the distribution of Rhipicephalus microplus and R. decoloratus in Zimbabwe. Vet. Parasitol. Reg. Stud. Rep. 14, 41–49. 10.1016/j.vprsr.2018.08.006 10.1016/j.vprsr.2018.08.006 31014735

[CR40] Marvelous Sungirai, Doreen Moyo, Patrick De Clercq, M. Madder, 2015, Communal farmers’ perceptions of tick-borne diseases affecting cattle and investigation of tick control methods practiced in Zimbabwe, Population structure of R.microplus isolates from Zimbabwe, 10.1016/j.ttbdis.2015.07.015 10.1016/j.ttbdis.2015.07.015 26234572

[CR41] Svinurai W, Mapanda F, Sithole D, Moyo EN, Ndidzano K, Tsiga A, Zhakata W (2017). Enteric methane emissions and their response to agro-ecological and livestock production systems dynamics in Zimbabwe. The Science of the Total Environment..

[CR42] Tavirimirwa, B., Mwembe, R., Ngulube, B., Banana, N. Y. D., Nyamushamba, G. B., Ncube, S., & Nkomboni, D., 2013. Communal cattle production in Zimbabwe: A review. Livestock for Research for Rural Development, 25(12), http://www.lrrd.cipav.org.co/lrrd25/12/tavi25217.htm

[CR43] Thomas, A., & Babu, M. N., 2020. Crowdsourcing Knowledge: An Extension Approach for Remunerative and Sustainable Home Garden Farming Systems in Kerala. Journal of Extension Education, 32(1). 10.26725/JEE.2020.1.32.6429-6440

[CR44] Emma Thomas, Mark Riley, Jack Spees 2019 Knowledge flows: Farmers’ social relations and knowledge sharing practices in ‘Catchment Sensitive Farming’

[CR45] UNICEF, Annual Report, 2021, UNICEF Zimbabwe

[CR46] Walker AR (2011). Eradication and control of livestock ticks: Biological, economic and social perspectives. Parasitology.

[CR47] Madhavi Wijerathna & W. A. D. P. Wanigasundera, 2020a “Communication” in the Context of Agricultural Extension: Past, Present and Way Forward in Achieving Sustainable Food Systems in Sri Lanka

[CR48] Wilkins EJ, Miller HM, Tilak E, Schuster RM (2018) Communicating information on nature-related topics: Preferred information channels and trust in sources. PLoS One 13(12): e0209013. 10.1371/journal.pone.0209013 10.1371/journal.pone.0209013 PMC6291159 30540834

[CR49] www.newzimbabwe.com/214958-2/Zimbabwe loses 15 000 cattle to tick-borne diseases, 18th April 2022

[CR50] www.thepatriot.co.zw/columns/eradicating-theileriosis-in-zimbabweurgent-intervention-required/ 11/03/ 2022

[CR51] www.zimfact.org/agriculture_in_zimbabwe/2018 March 12

[CR52] Zimbabwe, Ministry of Agriculture, Mechanization and Irrigation Development, Harare. https://fscluster.org/sites/default/files/documents/2nd_round_assessment_report_2020_draft_26_may.pdf

